# Solid State Stability and Kinetics of Degradation for Candesartan—Pure Compound and Pharmaceutical Formulation

**DOI:** 10.3390/pharmaceutics12020086

**Published:** 2020-01-21

**Authors:** Valentina Buda, Bianca Baul, Minodora Andor, Dana Emilia Man, Adriana Ledeţi, Gabriela Vlase, Titus Vlase, Corina Danciu, Petru Matusz, Francisc Peter, Ionuţ Ledeţi

**Affiliations:** 1 Faculty of Pharmacy, “Victor Babeş” University of Medicine and Pharmacy, 2 Eftimie Murgu Square, 300041 Timisoara, Romania; buda.valentina.oana@gmail.com (V.B.); corina.danciu@umft.ro (C.D.); 2 Faculty of Industrial Chemistry and Environmental Engineering, Politehnica University of Timișoara, Vasile Parvan Street 6, 300223 Timisoara, Romaniafrancisc.peter@upt.ro (F.P.); 3 Faculty of Medicine, “Victor Babeş” University of Medicine and Pharmacy, 2 Eftimie Murgu Square, 300041 Timisoara, Romania; andorminodora@gmail.com (M.A.); danaemilia@yahoo.com (D.E.M.); matusz@umft.ro (P.M.); 4 Research Centre for Thermal Analysis in Environmental Problems, West University of Timişoara, 300115 Timisoara, Romania; gabriela.vlase@e-uvt.ro (G.V.); titus.vlase@e-uvt.ro (T.V.)

**Keywords:** candesartan cilexetil, pharmaceutical formulation, decomposition, thermal analysis, kinetic study, isoconversional methods, Friedman method, Flynn–Wall–Ozawa method, NPK method

## Abstract

The aim of this work was to assess the impact of an excipient in a pharmaceutical formulation containing candesartan cilexetil over the decomposition of the active pharmaceutical ingredient and to comparatively investigate the kinetics of degradation during thermolysis in an oxidative atmosphere under controlled thermal stress. To achieve this, the samples were chosen as follows: pure candesartan cilexetil and a commercial tablet of 32 mg strength. As a first investigational tool, Universal attenuated total reflection Fourier transform infrared (UATR-FTIR) spectroscopy was chosen in order to confirm the purity and identity of the samples, as well as to check if any interactions took place in the tablet between candesartan cilexetil and excipients under ambient conditions. Later on, samples were investigated by thermal analysis, and the elucidation of the decomposition mechanism was achieved solely after performing an in-depth kinetic study, namely the use of the modified non-parametric kinetics (NPK) method, since other kinetic methods (American Society for Testing and Materials—ASTM E698, Friedman and Flynn–Wall–Ozawa) led to inadvertencies. The NPK method suggested that candesartan cilexetil and the tablet were degraded by the contribution of two steps, the main being represented by chemical degradation and the secondary being a physical transformation. The excipients chosen in the formulation seemed to have a stabilizing effect on the decomposition of the candesartan cilexetil that was incorporated into the tablet, relative to pure active pharmaceutical ingredient (API), since the apparent activation energy for the decomposition of the tablet was 192.5 kJ/mol, in comparison to 154.5 kJ/mol for the pure API.

## 1. Introduction

Candesartan is a selective, potent, and competitive antagonist of AT_1_ receptor (Angiotensin II receptor type 1) that is part of angiotensin II (Ang II) receptor blocker’s (ARBs) family of drugs [1,2]. Together with losartan (the first synthesized substance of the class), olmesartan, valsartan and irbesartan, they are commonly known as biphenyl-tetrazole ARBs. 

**Figure 1:**
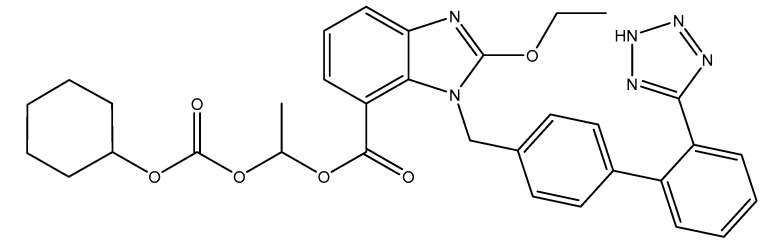
Structural formula of candesartan cilexetil (CC).

ARBs are small molecular weight pharmaceutical substances that modulate the rennin–angiotensin system and that have a high specificity for the AT_1_ receptor of angiotensin II. Via their mechanism of action (inhibitory pattern and dissociation rates form the receptor), they can be classified into surmountable (losartan, eprostan) or insurmountable ARBs (candesartan, irbesartan, valsartan, olmesartan, telmisartan and azilsartan) [2]. Candesartan is an oral, long-active, lipophilic compound that is available as a pro-drug; candesartan cilexetil (abbreviated CC; Figure 1) is used in formulations in order to increase their bioavailability. During gastrointestinal absorption (15% bioavailability), it is rapidly and completely transformed into the active metabolite of candesartan through a process of ester hydrolysis. It binds in a high percentage to plasmatic proteins (95%) and crosses the blood–brain barrier poorly. It can cross also the placental barrier. In its active form, candesartan is almost entirely excreted in feces (67%) and urine (33%) [3,4]. 

Acting in a dose-dependent manner, candesartan can be used in several pathologies such as essential arterial hypertension [5], ventricular hypertrophy [6], heart failure [7], diabetic nephropathy, [8] retinopathy [9], myocardial infarction [10], endothelial dysfunction [6,11], prehypertension [12], the prevention of atrial fibrillation [13], migraine prophylaxis [14], and stroke [15]. Recently, it has been shown to ameliorate brain inflammation associated with Alzheimer’s disease [16] and to induce neuroprotective effects in patients suffering from Parkinson’s disease [17]. Moreover, it has been shown to reduce inflammation and mucin production in patients with allergic asthma [18] and to induce benefits in a gestational hypertension mouse model [19].

CC is available in doses of 4, 8, 16 and 32 mg, and it is administered in a function of the pathology (usually 4 mg for the treatment of heart failure and 16 mg for the treatment of hypertension) once daily in monotherapy or in combination with other classes of drugs [1,3,4]. The most common side effects reported include hypotension, hyperkalemia, impaired renal function, and allergic reactions, although other adverse effects, such as dizziness, fatigue, headache, diarrhea, impotence, upper respiratory infections, rhabdomyolysis, and angioedema [20], have been reported. CC is considered to be teratogenic, and its use should be avoided in pregnant women because it might interfere with the rennin–angiotensin–aldosterone system and decrease fetal renal function (oligohydramnios condition). Moreover, it is not known whether it is excreted into human milk, so CC administration should be avoided in lactating women [1,20].

The thermal behavior of active pharmaceutical ingredients (APIs) as pure components in binary mixtures with excipients and in pharmaceutical formulations is of great importance in drug science [21,22,23,24,25,26]. Even if in the case of pharmaceuticals, the term “stability” is usually correlated with the loss of the active pharmaceutical ingredient from formulation, and “solid-state stability” can also designate the response of an API or a pharmaceutical formulation to thermal stress. However, in both cases, the decomposition of the API due to chemical processes or even physical transitions (phase transitions such as polymorphism and crystallization) in the presence of excipients dictates the shelf-life of the formulation [27,28]. Additionally, an adequate selection of excipients can lead to formulations with longer shelf-lives, as the presence of the excipients may have a stabilizing effect on the decomposition of APIs in formulation, relative to the same API as pure compound [29,30,31,32,33,34]. The literature is rather poor in presenting the physico-chemical characterization of candesartan and the prodrug CC. In 2014, Moisei et al. reported a compatibility study between several antihypertensive APIs and showed that CC is compatible with croscarmellose sodium, magnesium stearate and colloidal silica [35]. Amer et al. [36] realized a comparative evaluation of candesartan and CC by means of physicochemical properties and in vitro dissolution studies in order to replace the ester prodrug candesartan cilexetil (CC) by its active metabolite candesartan, and the results were promising. Tita et al. reported on the thermal behavior of candesartan, active substance and in pharmaceutical compounds in a dynamic nitrogen atmosphere in non-isothermal conditions, but they did so without mentioning any data regarding the strength of investigated pharmaceutical formulations and without clearly indicating if candesartan or candesartan cilexetil were studied as pure APIs; the paper mentions the cilexetil prodrug, but, though the chemical structure of the drug corresponds to candesartan, it does not correspond to the cilexetil derivative [37]. 

A successful development of novel cationic self-nanoemulsifying drug delivery systems of CC with improved biopharmaceutical profiles was realized by Sharma et al. and reported in 2015 [38], while Yuce et al. realized a full factorial experimental design of the immediate release of 32 mg CC tablets in 2016 [39] that also mentioned that CC possesses a low chemical stability; however, the latter did so without carrying out a kinetic study regarding the drug’s stability. Regarding the kinetic studies that have been carried out on CC, only the paper reported by Cui et al. [40] mentioned the kinetics of solvent-mediated polymorphic transitions by using Raman spectroscopy, and no study has been published regarding the solid state stability and kinetics of degradation for CC—both pure compound and pharmaceutical formulation.

Following the lack of data regarding the solid-state characterization of CC as a pure API, data regarding CC that is has been incorporated into a solid pharmaceutical formulation along with excipients, and evaluations of the kinetics of decomposition under thermal stress, we aimed to make a comparative investigation of a pure prodrug API and a commonly-used generic solid formulation containing 32 mg of the same API per tablet. The samples were initially investigated by Universal attenuated total reflection Fourier transform infrared spectroscopy (UATR-FTIR spectroscopy) in order to confirm the purity and identities of the API and also to obtain information regarding the compatibility of the API with the excipients that were used in the solid formulation in ambient conditions; these results were later completed with a search of interactions between the components during thermal stress. The comparative thermal behavior of CC vs. a tablet containing CC as API (CCTAB) was preliminary investigated by using the thermoanalytical data that were recorded for both samples in identical experimental conditions and later by an in-depth kinetic study consisting of the use of a classic kinetic method (American Society for Testing and Materials—ASTM E698 method) and that was completed with the isoconversional methods of Friedman (Fr) and Flynn–Wall–Ozawa (FWO). The decomposition mechanism was evaluated in the last part of the study by employing the modified non-parametric kinetics (NPK) method and focused mainly on the evaluation of the stabilizing/destabilizing excipient effect over the decomposition of CC in CCTAB relative to pure CC.

## 2. Materials and Methods

### 2.1. Samples and Preparation

Pure candesartan cilexetil Chemical Reference Substances (CRS), abbreviated CC, Batch 1.1, Id 003QK7), according to European Pharmacopoeia Reference Standard, was commercial product of European Directorate for the Quality of Medicines and Healthcare EDQM, Council of Europe (Strasbourg, France) and was used without further purification.

The pharmaceutical formulation (abbreviated CCTAB) was a tablet with a strength of 32 mg CC, which is the highest concentration available. This concentration was chosen in order to get resolute instrumental data. The tablet (of mass 260 mg) was crushed in an agate mortar with a pestle, homogenized for ten minutes, and then sieved. As excipients, the producers declared for CCTAB was the use of hydroxypropyl cellulose, calcium carboxymethyl cellulose, red iron oxide (E172), lactose monohydrate, starch from corn, macrogol 8000, and magnesium stearate.

### 2.2. Spectroscopic Description of Samples

FTIR spectra were recorded on Perkin Elmer SPECTRUM 100 device (Perkin-Elmer Applied Biosystems, Foster City, CA, USA) without an a priori preparation of the sample. The data were collected in a 4000–650 cm^−1^ domain on a UATR device in order to eliminate the effect of pelleting due to use of KBr and pressure. Spectra were built up after a number of 32 co-added scans. The spectral range 2300–1900 cm^−1^ represents the noise signal of the crystal and has no spectroscopic significance.

### 2.3. Thermal Stability Investigations

Thermal analysis investigations were carried out on a Perkin-Elmer DIAMOND apparatus (Perkin-Elmer Applied Biosystems, Foster City, CA, USA) for simultaneously obtaining the TG (thermogravimetric/mass curve), DTG (derivative thermogravimetric/mass derivative) and HF (heat flow curve) in dynamic air atmosphere (100 mL·min^−1^) while using open aluminum crucibles. The analyses were carried out under non-isothermal conditions at four heating rates β, namely 5, 7, 10 and 12 °C·min^−1^ from ambient temperature up to 400/500 °C. To determine the thermal effects, the Differential thermal analysis (DTA) data (in µV) were converted in HF (Heat Flow) data (in mW). The HF data (mW) were converted in normalized HF data by dividing the signal by mass of sample, thus obtaining the Differential scanning calorimetry (DSC) data (in mW·mg^−1^).

In order to assure the reproducibility of the thermal investigations, each analysis was carried out in duplicate, and the results were practically identical. 

### 2.4. Kinetic Investigations

The kinetic study (ASTM E698, Friedman and Flynn–Wall–Ozawa methods) was carried out on the main decomposition step that took place between 190 and 310 °C while using the AKTS Thermokinetics Software Version 5.2 (AKTS AG TechnoArk, Siders, Switzerland). The mathematical background and importance of using isoconversional kinetic methods have been extensively reported in the literature [41,42,43,44,45,46]. The decomposing mechanism of CC and solid pharmaceutical formulation CCTAB was evaluated by using the modified NPK method by a protocol that has been previously reported by our research group [47,48,49,50,51,52]. 

## 3. Results and Discussions

In order to confirm the identity and purity of CC, as well the presence of the API in the analyzed pharmaceutical formulation, FTIR spectra were drawn up by using the UATR technique. Some advantages of using the UATR technique are:

The samples can be analyzed without any preparation, as the technique is non-destructive and economical, since it requires small quantities of samples.

The required time for analysis is very short, since the salt pelleting that is used in classic FTIR technique is no longer required.

The technique does not require the preparation of a blank background, as in the case of pelleting technique.

The applied pressures on the surface of the ATR crystal are significantly lower than the ones used during pelleting, so polymorphic transitions and morphological modification of the analytes are not expected to occur.

### 3.1. Spectroscopic Description of Samples

**Figure 2:**
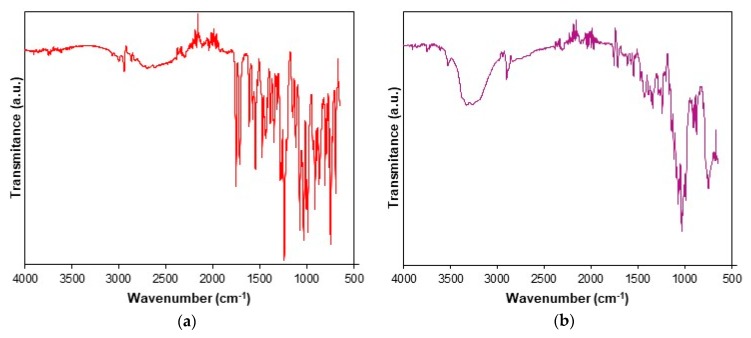
Universal attenuated total reflection Fourier transform infrared (UATR-FTIR) spectra recorded for analyzed samples: (**a**) CC and (**b**) tablet (CCTAB).

The UATR-FTIR spectra recorded for CC and CCTAB are presented in Figure 2.

The UATR-FTIR spectra recorded for CC revealed the presence of the following bands: aromatic and aliphatic CH stretching in the spectral range of 3070–2850 cm^−1^, with peaks at 3067, 3032, 2998, 2942 and 2863 cm^−1^; the stretching of the carbonyl C=O group from the asymmetric organic carbonate –OC(=O)O– moiety showed an intense band at 1751 cm^−1^; an ester carbonyl group appeared as an intense band at 1713 cm^−1^, this being characteristic for the polymorphic Form I of CC [53]. The bending of NH appeared at 1613 cm^−1^, and aromatic C–N stretching was visible at 1348 cm^−1^. The bands that appeared at 1240 and 1074 cm^−1^ were the consequences of the acyl–alkoxy tautomeric equilibrium from the R1–C(=O)O–R2 moiety. The C–O ether stretch was represented by the band at 1032 cm^−1^. The tetrazolyl moiety showed characteristic absorption bands in the 1640–1335 and 1200–900 cm^−1^ spectral regions. Due to extended conjugation of the tetrazolyl moiety with a biphenyl one, the vibrations of the С=С, С=N and N=N bonds were complex, so the individual assignment of bands to each bond could not be made. The absorption bands that appeared in the range of 1640–1335 cm^−1^ were due to the stretching, while the ones occurring in the 1200–900 cm^−1^ range were due to skeletal vibrations of the cycle, as well as by the combination bands. The data were assigned according to literature [54] and were in good agreement with previously reported studies [36,55,56,57].

The spectrum of CCTAB is more complex than that of pure API, since it contains more overlapping bands due to existence of excipients in the formulation. In the 3569–2989 cm^−1^ range, a broad band was visible due to the presence of OH groups in the structure of some excipients (hydroxypropyl cellulose, calcium carboxymethyl cellulose, lactose monohydrate, starch, and macrogol 8000), as well due to the water from lactose monohydrate. The characteristic bonds of CC were also present in the spectra of the tablet at the same wavenumbers or insignificantly shifted towards ± 3 cm^−1^, revealing that no interactions occurred under ambient conditions between the API and selected excipients.

### 3.2. Thermal Stability Investigations

**Figure 3:**
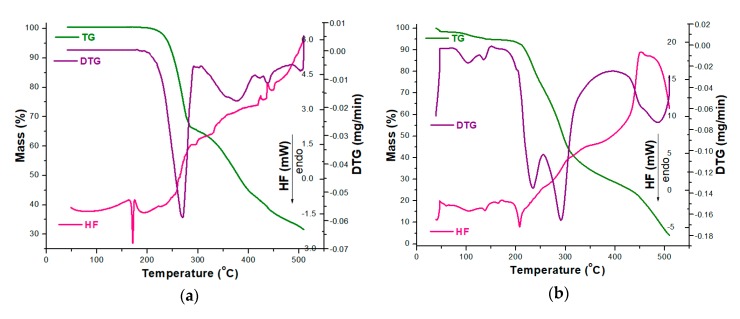
Thermoanalytical thermogravimetric/derivative thermogravimetric/heat flow (TG/DTG/HF) curves recorded in an oxidative synthetic air atmosphere at β = 5 °C·min^−1^ for analyzed samples: (**a**) CC and (**b**) CCTAB.

In order to analyze the stability of CC as a pure compound and in the presence of excipients, TG/DTG/HF curves were initially recorded in an oxidative atmosphere (dynamic air), and the obtained data are presented in Figure 3.

The thermoanalytical profile of CC revealed that it was thermally stable up to 164 °C, when mass loss occurred with decomposition along with melting, once again confirming the existence of the polymorphic Form I of CC, as previously suggested by the UATR-FTIR spectroscopy data [53]. The main mass loss process took place in the 164–310 °C range, with a considerable mass loss; Δ*m* = 35.66%. In this range, the HF curve revealed that melting occurred along with the decomposition, and the main process took place between 164 and 175 °C, with an HF_peak_ at 171 °C. Between 310 and 510 °C, the mass loss was 32.71% and was evidenced by several consecutive processes, as revealed by both the DTG and HF curves by peaks at 376, 420, 438 and 503 °C (DTG) and 424, 438 and 507 °C (HF). All the processes associated with mass loss were represented by thermo-oxidations, since the HF profile was ascending and the peaks corresponded to exoergic events (Figure 3a).

The tablet (CCTAB) presented a more complex pathway of decomposition (Figure 3b), since it contained, along with CC, a considerable amount of excipients. The tablet initially lost the water found in formulation in the temperature range of 35–51 °C (Δ*m* = 1.49%, endoergic event between 35 and 51 °C, HF_peak_ at 42 °C). After dehydration, the anhydrous composition of the tablet showed a narrow stability interval solely between 51 and 73 °C when the first decomposition occurred up to 164 °C due to the presence of excipients: Δ*m* = 3.77% and DTG peaks at 103 and 136 °C. In this range, the HF curve revealed several endoergic processes due to the melting of the excipients (peaks at 60 and 105 °C) and of the API (peak at 164 °C). The main decomposition process took place between 164 and 369 °C, with a corresponding mass loss Δ*m* = 65.73%, as evidenced by the two successive processes revealed by the DTG curve with maximums at 233 and 291 °C. In this range, the HF curve revealed an endothermic process (187–219 °C, HF_max_ at 208 °C). Later on, the last stage of decomposition (369–510 °C) led to a residual mass of 4.06%, and, with the advance of the thermal treatment, the exoergic process became more intense due to oxidative thermolysis (HF_max_ at 451°C and DTG_max_ at 486 °C).

### 3.3. Kinetic Investigations

The screening of compatibility between the components in a multi-component system is really difficult to achieve by classic thermal analysis because the overlapping of thermal events due to an excipient can hide the events associated with the degradation of an API. Based on this fact, a comparative kinetic study of thermal degradation of the pure API vs. the same API incorporated in an existing pharmaceutical formulation (or in multicomponent system with excipients) could lead to important information regarding the stability of the API, as well as its compatibility/incompatibility with the system components [58].

Since the thermal stability of an API is different from the one of a solid pharmaceutical formulation, an in-depth kinetic study can reveal if the excipients determine the increase or the decrease of stability of CC after its incorporation in a tablet. In a previous paper, we investigated the solid-state stability of perindopril erbumine as a pure API and of perindopril erbumine in a pharmaceutical formulation, and we showed that the used excipients (anhydrous colloidal silica, microcrystalline cellulose, lactose, and magnesium stearate) should be used in newly-developed generic solid pharmaceutical formulations because they contributed to an increased thermal stability of perindopril erbumine [58]. 

The kinetic analysis was realized based on the DTG data that were obtained for the thermolysis in an oxidative atmosphere of CC and CCTAB at four heating rates, namely β = 5, 7, 10 and 12 °C·min^−1^. 

As a first kinetic method, the ASTM E698 kinetic method was used. The ASTM E698 model is based on the Ozawa method, requires at least three experiments at different heating rates, and assumes that the extent of the reaction is a constant value independent of the heating rate when a DTG curve reaches its extreme value (DTG_peak_). The mathematical model of the ASTM E698 method is presented in Equation (1):

(1) $$\ln\beta =\mathrm{const}-1.052\cdot E_{a}\cdot R^{-1}\cdot T_{\max}^{-1}$$

where *β* is the heating rate (°C/min or equivalent K/min), *E_a_* is the apparent activation energy (kJ/mol), *R* is the gas constant, and *T_max_* is the absolute temperature (K) where extent of the reaction reaches its extreme value (corresponding to the DTG_peak_ from thermoanalytical DTG curve at each *β*). 

**Figure 4:**
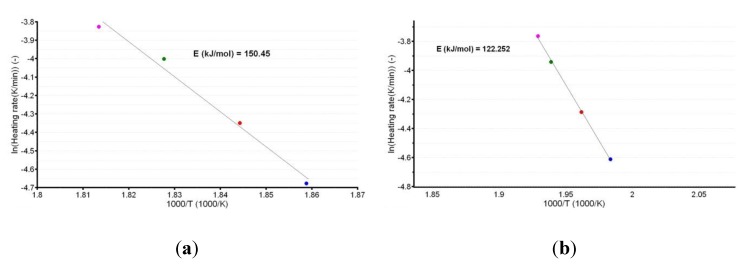
Linear plotting according to the American Society for Testing and Materials (ASTM) E698 kinetic method for CC (**a**) and CCTAB (**b**).

A plot of the natural logarithm of the heating rate vs. the peak temperature provides the information that is necessary to calculate the apparent activation energy (Ea), as shown in Figure 4a,b.

The linear dependencies obtained from the ASTM E698 kinetic method suggest a good correlation of the data, since the *R*^2^ values were 0.988 for CC and 0.999 for CCTAB. Additionally, the estimated values of *E_a_* (150.5 kJ/mol for CC and 122.3 kJ/mol for CCTAB) suggested good thermal stability for CC as a pure API, as well as in pharmaceutical formulation. However, a small destabilizing effect of the excipients was observed, since the value obtained for formulation was approximately 28.2 kJ/mol and thus inferior to that of pure CC. 

Since International Confederation for Thermal Analysis and Calorimetry (ICTAC) protocols recommend the use of both differential and integral isoconversional methods to gain an in-depth view of decomposition kinetics—two isoconversional methods, a differential one (Friedman), and an integral one (Flynn–Wall–Ozawa) were employed in this study. The advantages and limitations of isoconversional methods have been reported in the literature [59,60,61]. By far, the most important advantage of using isoconversional methods is the fact that they assure the estimation of the apparent *E_a_* with the advance of the reaction (expressed as conversion degree *α*) without assuming an approximation for the conversion function f(*α*). Additionally, by the analysis of variation of individual *E_a_* values that are obtained for each *α*, an indication for modification of degradation mechanism with the modification of thermal treatment is offered: iI each individual *E_a_* value that is determined for each *α* falls in the range of ${\bar{E}}_{a}\pm 0.1{\bar{E}}_{a}$, the decomposition mechanism is not influenced by the modification of the thermal treatment of the sample and is independent of *α*. If this premise is not true, the decomposition mechanism changes during the thermal treatment of the sample and is dependent of *α*.

**Figure 5:**
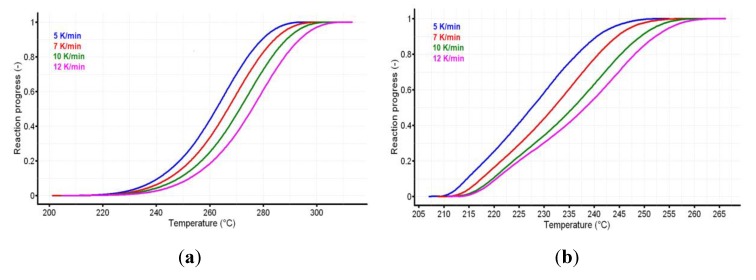
The reaction progress vs. temperature for (**a**) CC and (**b**) CCTAB.

**Figure 6:**
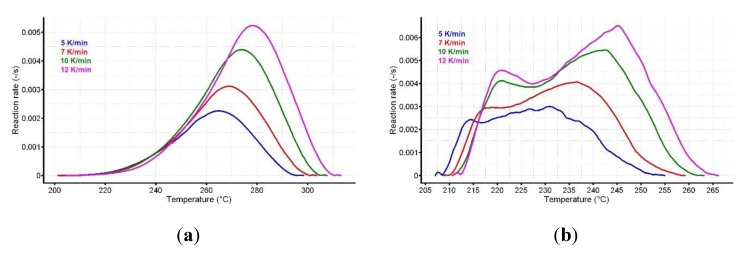
The variation of reaction rate vs. temperature for (**a**) CC and (**b**) CCTAB.

The reaction progress vs. temperature is presented in Figure 5a,b for the active substance and the pharmaceutical formulation. The variation of reaction rate vs. temperature for CC and CCTAB is presented in Figure 6a,b. 

The analysis of variation of reaction rate vs. temperature may suggest different decomposition mechanisms for pure CC and CCTAB; in the last case, this difference may have been due to the presence of excipients. The mathematical development of isoconversional methods, as well their deduction, were reported earlier [58,62]. The Friedman method (Fr) was used in the linearized form [63], as shown in Equation (2).

ln (*β*·d*α*/d*T*) = ln [*A*·f(*α*)] − *E_a_*·*R*^−1^·*T*^−1^ (2)

where *T* is the absolute temperature (K) and *A* is the pre-exponential factor, as suggested by the Arrhenius equation. 

**Figure 7:**
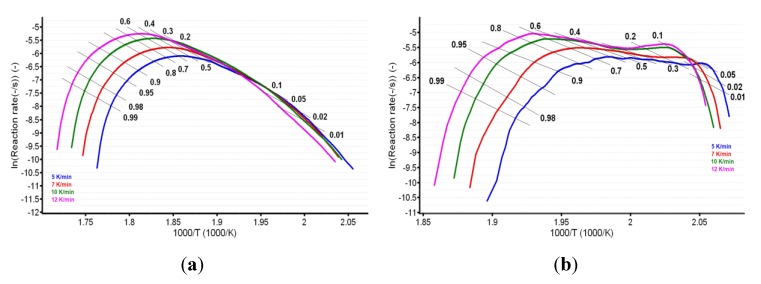
Plotting of Friedman method at selected four heating rates for (**a**) CC and (**b**) CCTAB.

For known *α* at the selected heating rates, the plot ln (*β*·d*α*/d*T*) vs. (1/*T*) was linear. The values of the apparent activation energy (*E_a_*) for the two samples were obtained (Table 1) by evaluating the slopes of these straight lines (see Figure 7a,b).

The integral isoconversional method of Flynn–Wall–Ozawa (FWO) [64,65,66,67] was used in the linearized form presented in Equation (3):

ln *β* = ln [*A*·*E_a_*·*R*^−1^·g^−1^(*α*)] − 5.331 − 1.052·*E_a_*·*R*^−1^·*T*^−1^ (3)

where g(*α*) is the integral conversion function.

**Figure 8:**
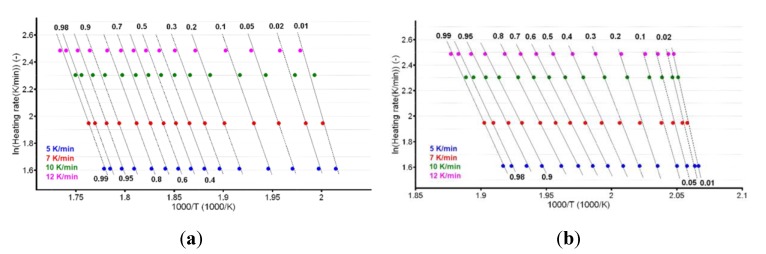
Plotting of Flynn–Wall–Ozawa method at selected four heating rates for (**a**) CC and (**b**) CCTAB.

The plotting of ln*β* vs. T^−1^ allowed for the estimation of apparent activation energy values (*E_a_*) for each conversion degree. The obtained results are presented in Figure 8a,b and Table 1.

In the case of CC, both isoconversional methods indicate that the decomposition mechanism of the substance during thermolysis was unique and independent of the conversion degree and heating rate, since the values of individual *E_a_* values were around the mean value (155.5 kJ/mol) and inside the ± 15 kJ/mol interval (corresponding to confidence interval range ${\bar{E}}_{a}\pm 0.1{\bar{E}}_{a}$), namely between 149.0 kJ/mol (at *α* = 30%) and 162.8 kJ/mol (at *α* = 95%), according to the Friedman method. The same trend was observed in the case of the integral method of FWO, where the variation was between 154.7 kJ/mol (at *α* = 40%) and 168.5 kJ/mol (at *α* = 5%), falling in the confidence interval around the mean value of 157.7 kJ/mol (Table 1). Additionally, the results are in good agreement with the ASTM E698 method, as previously discussed.

**Figure 9:**
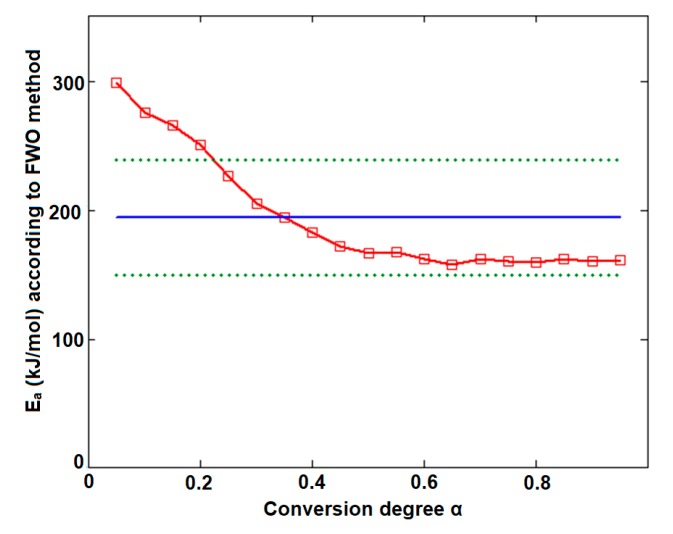
Plotting of Flynn–Wall–Ozawa method at selected four heating rates for CCTAB.

In the case of CCTAB, the results indicated by the differential and integral method were not in good agreement, since the variation between the mean values was higher than 37 kJ/mol, clearly suggesting a complex pathway of decomposition. The particular values *E_a_* vs. *α* according to the Fr method did present a randomized trend with increases and decreases as the reaction advanced, so the maximum value was almost double the minimum (215.7 kJ/mol at *α* = 15% vs. 112.7 kJ/mol at *α* = 35%). In this case, the mean *E_a_* value of 157.7 kJ/mol was only calculated for a comparison to the result suggested by the FWO method and not to characterize the decomposition mechanism of the sample during thermal treatment, because, in this case, a modification of the mechanism during thermolysis occurred. The data suggested for the FWO method also clearly indicated the dependency of the mechanism of decomposition with the thermal treatment of the sample. The variation of the apparent *E_a_* fell between 158.8 kJ/mol (*α* = 70%) and 307.3 kJ/mol at the beginning of the decomposition process (*α* = 5%). Most of the values *E_a_* vs. *α* were outside the confidence interval, and the mean value was in discrepancy with the results that were preliminarily obtained by the ASTM E698 method. The results obtained by the FWO method for CCTAB, namely the decrease of *E_a_* with the advance of reaction, can be explained by the variation of the parameters of activation during the heating process due to the complexity of the heterogeneous process and/or due to the influence of mass transfer and heat transfer over the reaction rate [68]. Vyazovkin et al. associated the trend and aspect of the *E_a_* vs. *α* curve with the mechanism of complex heterogenous processes, leading to important qualitative information over the thermolysis—so, for a variation like decreasing of *E_a_* vs. *α* (Figure 9), a process with reversible step is suspected [69,70,71,72,73].

Additionally, the considerable dissimilarities suggested by the Fr and FWO methods in the kinetic analysis of decomposition of CCTAB can be explained by the integral processing of the data in the case of integral method of FWO, in comparison to Friedman method, where the data processing is differential [58,62].

Since the results of kinetic studies (classic ASTM E698 method and isoconversional ones) were in disagreement, a fourth method was employed in the study, namely the modified NPK method. Initially, the NPK method was developed in 1998 through the studies of J. Sempere, R. Nomen and R. Serra [74,75,76] and carried on in a kinetic study on DSC data in isothermal conditions without using an a priori model for the k(*T*), nor for f(*α*), and only accepting the axiom that the reaction rate (*r*) can be expressed as a product of two separable functions—one dependent solely on temperature and one dependent solely on conversion, as shown in Equation (4):

(4) $$r=\frac{d\alpha }{dt}=k\left(T\right)\cdot f\left(\alpha \right)$$

where k(*T*) is the rate constant and f(*α*) is the differential conversion function. 

Regarding the direct consequence for assuming the validity of this axiom (that is unanimously accepted in chemical kinetics), it worth mentioning two, namely the fact that the reaction rate can be represented as a 3D surface (Π), and that the reaction rate can be expressed as a product of matrices. 

**Figure 10:**
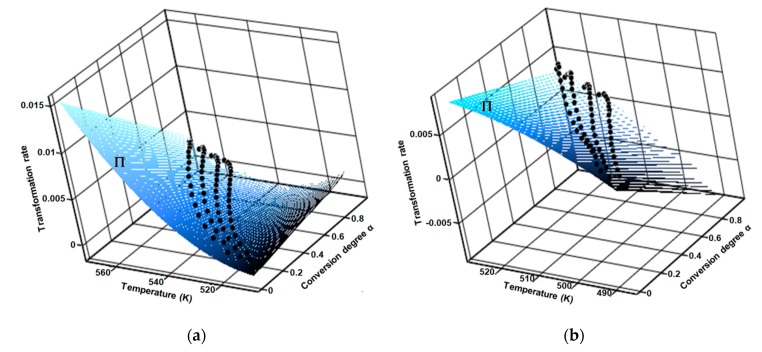
Surface of fitted 3D reaction rate generated by the interpolation of family of curves obtained at four selected heating rates for (**a**) CC and (**b**) CCTAB.

Regarding the aforementioned first consequence by carrying the thermoanalytical experiments at several heating rates (in this case four), the results was a family of curves, by which interpolation generated the fitted 3D surface of the reaction rate (Π), as seen in Figure 10.

Based on the validity of the axiom of kinetics presented in Equation (4), the modified NPK method was applied to the same data set as the previously employed kinetic methods. As a consequence, the reaction rates for the measurements at four different *β* values can be written as a matrix with *n* × *m* elements whose rows correspond to the constant value of the conversion degree, and the columns correspond to the different but constant values of temperature. By using the singular value decomposition algorithm (SVD) [77], the corresponding matrix was decomposed into two vectors, leading to the separation of the two theoretically assumed functions, namely the temperature-dependent one (as *k*(*T*)) and the conversion-dependent one (as f(*α*)). 

For the mathematical modelling of the two functions, any dependency can be theoretically used. However, for kinetics, the temperature dependence k(*T*) is usually represented by an Arrhenius model, while for the conversion function f(*α*), the model proposed by Šesták and Berggren is usually used [78], as seen in Equation (5):

(5) $$f\left(\alpha \right)=\alpha^{m}\cdot {\left(1-\alpha \right)}^{n}$$

where *m* and *n* are specific parameters for the investigated transformation process, with *m* corresponding to physical transformations (like phase transitions, crystallization, and diffusion) and *n* corresponding to a chemical transformation (decomposition, dehydrations, condensation, oxidation, etc.). 

The Šesták–Berggren model is a generalization of the conversion functions that were previously proposed and validated in literature, assuring the generality and universality of the modified NPK method and providing an advantage that can separate the physical and chemical events that occur during the transformation of a certain compound under thermal stress.

If a mass loss process evidenced by a TG curve consist in at least two simultaneous independent steps, then the apparent global rate of transformation (*r*_global_) is the sum of individual rates (*r*_1_, *r*_2_, *r*_3_…*r_z_*), as long as the degradation process consists in *z* individual steps Equation (6):

(6) $$r_{\mathrm{global}}=\sum_{i=1}^{z}r_{i}$$

and as a consequence, Equation (4) can be rewritten as Equation (7):

(7) $$r_{\mathrm{global}}=\sum_{i=1}^{z}k_{i}\left(T\right)\cdot f_{i}{\left(\alpha \right)}_{\;}$$

This type of data processing is leading to *z* sets of kinetic parameters (*E_a_*, *A*, *m*, *n*) and each process have a partial contribution to the global kinetic process, expressed as explained variance, *λ*.

**Figure 11:**
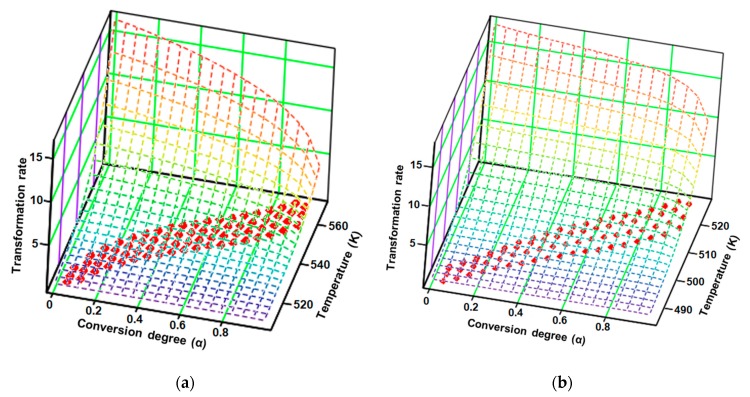
Experimental points (red dots) and the surface reaction generated by the kinetic parameters according to the modified non-parametric kinetics (NPK) method for (**a**) CC and (**b**) CCTAB.

Based on the mathematical model of the modified NPK method, the obtained results are systematized in Table 2, and the transformation rate surfaces in 3D dimensions with the coordinates (*β*·d*α*/d*T*; *α*; *T*) are presented in Figure 11. 

The results obtained after employing the modified NPK method revealed that both samples, CC and CCTAB, were degraded by the contribution of the two distinct steps. However, these degradation was done via different mechanisms. In each case, the main step (characterized by the explained variances of 87.0% and 75.3%, respectively) were due to chemical degradation, but they each had different reaction orders (*n* = 1/3 for CC and *n* = 1/4 for CCTAB), suggesting different mechanisms as the most important contribution to the degradation process (for CC, the partial *E_a_* value due to this step was around 141.6 kJ/mol, while it was 149.2 kJ/mol for CCTAB). The second process was represented by a physical transformation (since *n* = 0 and *m* ≠ 0), which can be attributed to the solid–liquid phase transition due to the melting of the API in both cases (HF_peak_ at 164 °C). In the case of CCTAB, the value of *m* changed in comparison to CC, as expected, since the HF curve revealed two more endoergic processes in this case due to the melting of excipients (peaks at 60 and 105 °C), along with the peak corresponding to the API. For both samples, the correlations obtained for the coefficient of determination for each step were excellent, thus proving the robustness of the kinetic model.

## 4. Conclusions

In this paper, a comparative thermal stability analysis was carried out between candesartan cilexetil as a pure active pharmaceutical ingredient and a commercial pharmaceutical formulation containing 32 mg of candesartan cilexetil per tablet. Initially, the samples were characterized by UATR-FTIR spectroscopy in order to check the identity and purity of the API and to investigate the possibly occurring interactions between the API and the excipients from the solid formulation. The results of the FTIR study confirmed the purity of CC and revealed that no interactions occurred between the API and the excipients from the tablet. 

As second investigational tool, thermal analysis was chosen. Thermoanalytical curves TG/DTG/HF were drawn up at four different heating rates, and, initially, the thermal behavior of the two samples was investigated and discussed as observed at *β* = 5 °C/min. Due to the compositional complexity of the tablet, its thermoanalytical profile differed significantly to the one of API, though this profile did reveal some of the characteristics of CC.

Since the kinetic study led to some inadvertencies regarding the stability of the API in the tablet in comparison to CC, four methods were employed to analyze the decomposition behavior of both samples, starting with classic methods (ASTM E698) and finishing with the modified NPK method, the later one providing important information regarding thermolysis, including the mechanism of decomposition. 

In comparison to the kinetic results suggested by previously-chosen methods (ASTM E698, Fr and FWO), the ones offered by NPK were in agreement for CC in all cases; for CCTAB, the results confirmed the data suggested by the FWO method, namely a stabilizing effect on the decomposition of CC in CCTAB relative to CC due to the presence of excipients in pharmaceutical formulation. The NPK method suggested that both samples were degraded by the contribution of two steps, the main step being chemical degradation and the secondary step being a physical transformation. The excipients chosen in the formulation seemed to have a stabilizing effect, since the apparent activation energy for decomposition of the tablet was 192.5 kJ/mol, and the apparent activation energy for decomposition of pure API was 154.5 kJ/mol. 

## Figures and Tables

**Table 1:** Apparent activation energies values apparent activation energy (*E_a_*) vs. conversion degree (*α*) obtained by the isoconversional methods and the ${\bar{E}}_{a}$ value.

Conversion Degree *α*	E_a_ (kJ/mol) vs. *α* for			
	CC		CCTAB	
	Fr	FWO	Fr	FWO
0.05	152.9	168.5	197.0	307.3
0.10	153.1	163.3	215.0	278.5
0.15	152.1	160.3	215.7	263.6
0.20	151.0	158.5	179.2	248.4
0.25	151.3	157.1	133.1	227.6
0.30	149.0	156.0	120.6	208.1
0.35	149.2	155.1	112.7	192.1
0.40	153.6	154.7	125.1	181.0
0.45	155.8	154.9	126.3	173.6
0.50	156.8	155.2	134.5	168.0
0.55	157.3	155.5	140.6	164.6
0.60	158.0	155.9	137.9	161.8
0.65	158.5	156.3	144.8	159.6
0.70	158.9	156.7	159.6	158.8
0.75	158.6	157.0	176.1	160.2
0.80	158.5	157.4	166.4	161.7
0.85	157.8	157.7	161.1	161.7
0.90	158.8	157.9	149.2	160.3
0.95	162.8	158.6	201.3	163.7
${\bar{E}}_{a}$ **(kJ/mol)**	**155.5 ± 0.9**	**157.7 ± 0.7**	**157.7 ± 7.2**	**194.8 ± 10.5**

**Table 2:** The results of kinetic analysis after employing the modified NPK method.

Sample	Step	*λ* (%)	*A* (s^−1^)	*E*_a_ (kJ/mol)	*n*	*m*	R^2^	f(*α*)	${\bar{E}}_{a}$ (kJ/mol)
CC	1	87.0	1.6 × 10^16^ ± 2.1 × 10^4^	162.8 ± 9.1	1/3	0	0.996	(1 − x)^1/3^	154.5 ± 11.1
	2	8.5	8.6 × 10^13^ ± 4.4 × 10^9^	147.2 ± 2.0	0	1/3	0.996	x^1/3^	
CCTAB	1	75.3	9.6 × 10^20^ ± 4.5 × 10^6^	198.2 ± 11.5	1/4	0	0.993	(1 − x)^1/4^	192.5 ± 16.6
	2	24.5	4.6 × 10^18^ ± 7.8 × 10^8^	176.1 ± 5.1	0	5/3	0.999	x^5/3^	
